# Recurrent amebic liver abscesses over a 16-year period: a case report

**DOI:** 10.1186/s13104-016-2275-0

**Published:** 2016-10-18

**Authors:** D. Creemers-Schild, P. J. J. van Genderen, L. G. Visser, J. J. van Hellemond, P. J. Wismans

**Affiliations:** 1 Institute for Tropical Diseases, Harbour Hospital, Rotterdam, The Netherlands; 2 Department of Infectious Diseases, Leiden University Medical Center, Leiden, The Netherlands; 3 Department of Medical Microbiology and Infectious Diseases, Erasmus University Medical Center and Institute for Tropical Diseases, Rotterdam Harbour Hospital, MC, Room Na-901K (Secr. Office), P.O. Box 2040, 3000 CA Rotterdam, The Netherlands

**Keywords:** *Entamoeba histolytica*, Amebiasis, Carrier state environment, Immune response, Relapse, Treatment

## Abstract

**Background:**

Amebic liver abscess is a rare disease in high-income countries. Recurrence of amebic liver abscess is even rarer with only a few previous reports. Here we present a patient who developed three subsequent amebic liver abscesses over a sixteen-year period.

**Case presentation:**

A Caucasian male developed recurrent amebic liver abscesses, when aged 23, 27 and 39 years. Only on the first occasion did this coincide with a recent visit to the tropics. The patient received adequate treatment during each episode. Possible explanations are persistent asymptomatic carrier state, cysts passage in his family, re-infection or chance.

**Conclusion:**

We describe the unusual case of a healthy male who developed recurrent amebic liver abscesses over a long period despite adequate treatment. Possible pathophysiological explanations are explored.

## Background

Intestinal amebiasis and amebic liver abscess, caused by the protozoan *Entamoeba histolytica,* are rarely reported from high-income countries. It is mostly encountered as an imported disease from regions with poor sanitation levels. Infection occurs through ingestion of *E. histolytica* cysts in contaminated food or water. Most infections remain asymptomatic while approximately 10 % of patients develop invasive disease and less than 1 % of the infections result in liver abscess [1]. Recurrence of amebic liver abscess after appropriate treatment with the combination of a nitroimidazole derivate with a luminal agent is rare with only a few previous reports [2–4]. The majority of those recurrences could be explained by failure to administer an adequate luminal agent [5–8]. We report an extraordinary case of a Caucasian (Dutch) male patient, who developed amebic liver abscesses three times over a period of 16 years, despite adequate combination treatment on each occasion.

## Case presentation

A previously healthy 23-year old, Caucasian man was admitted for the first time 19 years ago (1997), at the age of 23 with a 4-week history of upper right abdominal pain, loose stools and fever. Three months previously he had made a 5-week trip through Indonesia, Thailand and Malaysia. Laboratory testing revealed elevated inflammatory parameters and a slightly increased gamma-glutamyltransferase (Table 1). Ultrasound of the abdomen demonstrated a vaguely defined area in the right liver lobe compatible with an abscess and *Entamoeba* spp. cysts were detected in a stool sample, supporting the diagnosis of an amebic liver abscess. On admission no specific antibodies against *E. histolytica* were detectable using an ELISA method. The liver abscess was not aspirated. The patient was treated with metronidazole followed by diloxanide furoate with good clinical response. Serology for *E. histolytica* was positive one month later. Six months later, stool examination by microscopy for cysts was negative.

**Table 1:** Clinical characteristics of the 3 episodes of amebic liver abscesses

	1st episode (1997)	2nd episode (2001)	3rd episode (2013)
Age (years)	23	27	39
Symptoms	4 weeks fever, sweating, upper right abdominal pain	5 days spiking fever, malaise	5 days spiking fever, sweating, upper abdominal pain
Physical examination	Temperature 38.4, upper right abdominal tenderness	No abnormalities	temperature 37.1, upper abdominal tenderness
Laboratory tests (reference range)			
Hemoglobin mmol/L (8.5–11.0)	7,2	8,3	8,7
Leucocytes × 10^9^/L (4.0–10.0)	11	19,7	24,4
C-reactive protein mg/L (0–5)	ND	276	339^b^
Erythrocyte sedimentation rate mm/hr (<15)	94	63	74
Bilirubin total µmol/L (0–17)	8	29	15
Aspartate aminotransferase U/L (0–35)	23	19	28^b^
Alanine aminotransferase U/L (0–45)	69	46	23
Alkaline phosphatase U/L (0–390)	ND	107	99
Lactate dehydrogenase U/L (0–248)	244	288	160
Gamma-glutamyltransferase U/L (0–55)	190	58	63^b^
Creatinine µmol/L (64–104)	ND	101	86
Radiology	Liver abscess in the right lobe, size 6 × 5 cm (ultrasound)	Abscess high in the liver, size 10 cm (CT)	Liver abscess in the right lobe, size 4.9 × 4.5 cm with satellite abscesses (CT)^b^
Additional tests			
Stool microscopy	*Entamoeba* spp. Cysts	Negative	*Entamoeba* spp. cysts and trophozoites
Stool PCR *E. histolytica*	ND	ND	positive
Serology for amebiasis	<1:40, 1 month later 1:320	1:320	1:640 and 1:640^a^
Abscess fluid	ND	Culture negative, no amebic trophozoites	ND
Treatment	Metronidazole 750 mg tid 7 days	Metronidazole 750 mg tid 10 days	Metronidazole 750 mg tid 10 days
	Diloxanide furoate 500 mg tid 10 days	Diloxanide furoate 500 mg tid 10 days	Paromomycin 500 mg tid 10 days
		Aspiration and drainage liver abscess	

*CT* computed tomography, *PCR* polymerase chain reaction, *ND* not determined

^a^Results from samples collected June 9, 2013 and September 4, 2013, respectively (cut off <1:40)

^b^One week after presentation during the 3rd episode the patient was readmitted and C-reactive protein decreased to 46 mg/L, aspartate aminotransferase increased to and 42 U/L and gamma-glutamyltransferase increased to 129 U/L, the size of the abscess increased to 5 × 7 cm

^c^State of the art treatment on all occasions: nitroimidazole derivate followed by a luminal agent

Four years later (2001) the patient was admitted with a 10 cm large abscess high in the liver with a 5-day history of spiking fever and malaise. Since 1997 he had travelled once to Nepal and Bangladesh in 1999. The patient was again diagnosed with an amebic liver abscess, which was supported by positive serology results. Treatment was initiated with metronidazole. However, fever persisted and after 5 days percutaneous drainage of the abscess was performed. In the aspirate no trophozoites of *E. histolytica* were found and the bacterial cultures remained negative. The recovery hereupon was uneventful and a 10-day course of metronidazole was followed by diloxanide furoate for 10 days. Ultrasound of the liver demonstrated complete resolution of the abscess 6 months later.

**Fig. 1:**
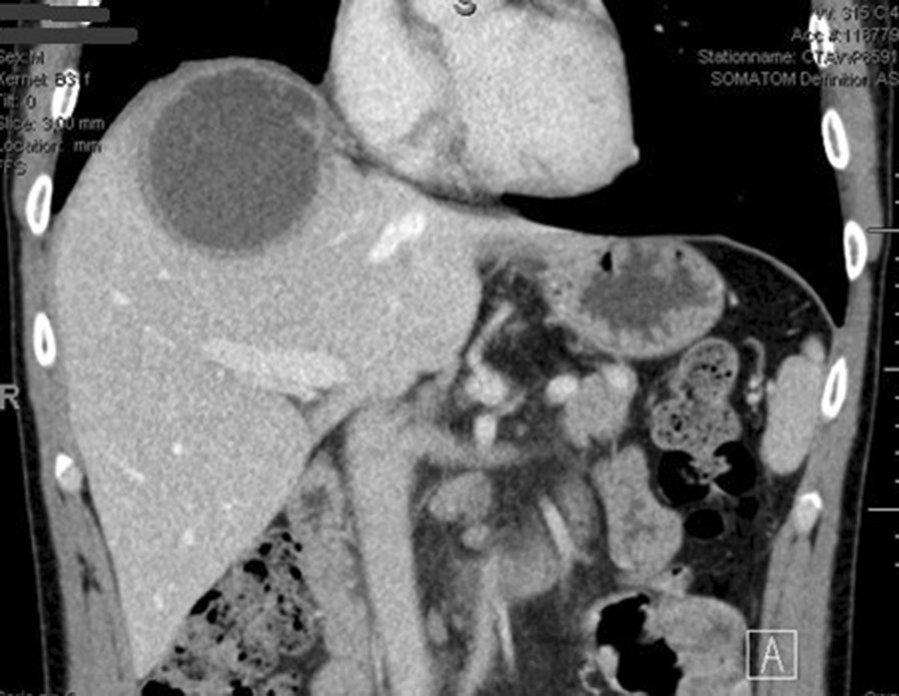
Upper abdominal computed tomography scan revealing an abscess of 7 cm in the right liver lobe with a small margin of liver tissue to the right hemidiaphragm and subcapsular extension

Finally, three years ago—with a twelve year interval (2013)—the patient was admitted for the third time with a 5-day history of spiking fever and upper abdominal pain. Two years earlier, in 2011, the patient had been in Thailand for one week and had made short trips to China (in 2006, 2009 and 2012). Laboratory examination revealed increased levels of inflammation markers and computed tomography (CT) showed an abscess of 5 cm in the right liver lobe with small satellite abscesses. For the third time the diagnosis of amebic liver abscess was made, based on positive serology results and the detection of *E. histolytica* in a stool sample by polymerase chain reaction (PCR). Treatment was started with metronidazole. Initially the patient responded well and was discharged on day 5. After 10 days of metronidazole, paromomycin was started. One week after discharge, the patient was readmitted with persistent low-grade fever and abdominal pain. C-reactive protein concentration had decreased (46 mg/L), but slightly increased levels of aspartate aminotransferase (42 U/L) and gamma-glutamyltransferase (129 U/L) were noted. A new CT-scan revealed a subtle increase in the size of the abscess with subcapsular extension (Fig. 1). Percutaneous drainage was deferred because the clinical condition improved. The patient subsequently made an uncomplicated recovery and has remained in excellent condition to date. There were no indications of immune deficiencies. Normal amounts of T- and B-cells, immunoglobulins and complement factors were found (Table 2). In addition a human immunodeficiency virus (HIV) test was negative.

**Table 2:** Laboratory evaluation of the immune system of the patient

Variable	Reference range adults	Results
B-cells (×10^9^/L)	0.10–0.40	0.27
T-cells (×10^9^/L)	0.70–1.90	1.50
CD4 (×10^9^/L)	0.40–1.30	0.90
CD8 (×10^9^/L)	0.20–0.70	0.56
NK-cells (×10^9^/L)	0.10–0.40	0.29
IgG (g/L)	7.00–16.00	11.10
IgG1 (g/L)	4.90–11.40	9.25
IgG2 (g/L)	1.50–6.40	2.60
IgG3 (g/L)	0.20–1.10	0.51
IgG4 (g/L)	0.08–1.40	0.55
IgA (g/L)	0.76–3.91	2.33
IgM (g/L)	0.45–2.30	1.46
C3 (g/L)	0.90–1.80	1.11
C4 (g/L)	0.10–0.40	0.20
C1Q (g/L)	0.05–0.25	0.24
M-protein		negative

Stool samples of the patient’s wife, who accompanied him from the first amebic abscess episode onwards, and their 2 children were investigated for *E. histolytica*. The wife tested positive for *E. histolytica* by PCR examination of the stool, but *E. histolytica* serology was negative. She was treated with paromomycin 500 mg tid for 10 days.

## Discussion

Our patient suffered from recurrent amebic liver abscesses on three separate occasions over a period of 16 years. Each time the diagnosis of amebic liver abscess was based on the clinical presentation, ultrasound or CT imaging and positive *E. histolytica* serology. The first and third episode *Entamoeba* spp. cysts were detected in stool samples. The abscess was aspirated only in the second episode; no trophozoites of *E. histolytica* were detected. The material was obtained 5 days after initiation of therapy, which probably accounts for the negative result of microscopy. The diagnosis of amebic liver abscess was further supported in each episode by clinical response after appropriate treatment.

Only a small proportion of *E. histolytica*-infected individuals develop invasive disease, whereas the majority of individuals carry the parasite within the gut without clinical symptoms [1]. Previous studies have indicated that both cell-mediated immunity and macrophage-mediated effector mechanisms are involved in host resistance to *E. histolytica* infection [9]. Neutrophils activated by interferon-γ (IFN-γ), tumour necrosis factor-α (TNF-α), or lipopolysaccharides (LPS) have amebicidal activity in vitro by releasing reactive oxygen species. Macrophages are also amebicidal after stimulation with IFN-γ or TNF-α. Important for the host defence against *E. histolytica* is a proper functioning of natural killer T cells (NKT cells) for the production of sufficient IFN-γ and nitric oxide, which induces the amebicidal activity of macrophages [9–11]. Moran et al. suggested that defects in production of reactive oxygen species are a risk factor for recurrence of amebic liver abscess [12]. There is also a protective role for lectin signalling and specific human leukocyte antigen (HLA) II alleles. A study of children in Bangladesh suggested a potential protective association with the HLA class II allele DQB1*0601 and the heterozygous haplotype DQB1*0601/DRB1*1501 for invasive amebiasis [13].

There is no evidence of immune deficiencies in our patient, as the absolute number of NKT cells and other immune cells was within the normal range with an otherwise uneventful medical history.

In contrast to amebic colitis, amebic liver abscess occurs more often in adult males. It is 10 times as common in men as in women and is a rare disease in children [1, 14]. Lotter et al. investigated in a mouse model for amebic liver abscess the role of female and male sexual hormones. Removal of testosterone by orchiectomy reduced the size of abscesses in male mice, while substitution of testosterone in female mice increased the size of amebic liver abscesses. This may be attributable to the inhibitory effect of testosterone on the IFN-γ production by NKT cells [15].

There are several possible explanations for the recurrence of amebic liver abscesses in this patient. The patient had travelled frequently to Asia, and therefore he may have simply been re-infected during his travels. Alternatively, recurrent disease may have occurred due to transmission of cysts between household members. Transmission of *E. histolytica* within households has been reported earlier [16]. The patient’s wife tested positive for *E. histolytica* cysts in the stool when investigated after her husband’s third episode. After 2002 she had travelled annually to China (Beijing) and had accompanied her husband during travel to Thailand and Nepal. A repeated infection from household members could have been supported by genotyping the strain(s) by means of PCR-fingerprinting [17]. Unfortunately, no material from past episodes was available to test this hypothesis. Persistent asymptomatic carrier state appears to be highly unlikely since the patient was treated appropriately for persisting intraluminal cysts on all occasions. In addition, the intervals would be exceptionally long for recurrences resulting from inadequate treatment which usually occur within months, not years. On the other hand, Blessmann et al. showed that diloxanide furoate was less effective than paromomycin in eradicating *E. histolytica* in asymptomatic cyst carriers [18]. Some form of immune dysfunction or genetic susceptibility could also have played a role, but this possibility seems unlikely. Obviously all speculation on the mechanism(s) involved remains hypothetical and this remarkable patient history may ultimately simply have been a matter of chance.

Of note in this case is the decision during the third episode to continue conservative treatment without drainage. In a recent Cochrane review there was no benefit for drainage in addition to metronidazole in case of uncomplicated amebic liver abscess [19]. Aspiration should be considered for patients with either a large left-lobe abscesses, a lack of clinical response within 5 days and in case of uncertainty about the diagnosis [1]. Our patient had a right lobe abscess of 7 cm—with a suspicion of subcapsular expansion - but with a clear margin to the right pleural cavity (Fig. 1). His clinical condition improved during metronidazole treatment, illustrated by the disappearance of the spiking fever and declining C-reactive protein levels (339–46 in 2 weeks).

## Conclusion

We present the unusual case of a healthy Dutch male with recurrent amebic liver abscesses on three separate occasions over a period of 16 years despite adequate treatment. While the exact nature of the underlying pathophysiological mechanism(s) will remain obscure, we explored some of the possibilities such as cysts from an asymptomatic close contact or an as yet unidentified immune deficiency in the host. Our observation serves as a welcome reminder of the unpredictable course of amebiasis even in the absence of recent travel to tropical regions.

## Abbreviations

CT: computed tomography
PCR: polymerase chain reaction
HIV: human immunodeficiency virus
IFN-γ: interferon-γ
TNF-α: tumour necrosis factor-α
LPS: lipopolysaccharides
NKT cells: natural killer T cells
HLA: human leukocyte antigen
